# Exploring Innovations in Human Milk Analysis in the Neonatal Intensive Care Unit: A Survey of the United States

**DOI:** 10.3389/fnut.2021.692600

**Published:** 2021-09-03

**Authors:** Stacey R. Ramey, Stephanie Merlino Barr, Katie A. Moore, Sharon Groh-Wargo

**Affiliations:** ^1^MetroHealth Medical Center, Case Western Reserve University, Cleveland, OH, United States; ^2^School of Medicine, Case Western Reserve University, Cleveland, OH, United States

**Keywords:** human milk, human milk analysis, individualized fortification, nutrition, neonatal ICU

## Abstract

**Introduction:** Human milk (HM) is the ideal enteral feeding for nearly all infants and offers unique benefits to the very low birthweight (VLBW) infant population. It is a challenge to meet the high nutrient requirements of VLBW infants due to the known variability of HM composition. Human milk analysis (HMA) assesses the composition of HM and allows for individualized fortification. Due to recent U.S. Food and Drug Administration (FDA) approval, it has relatively recent availability for clinical use in the US.

**Aim:** To identify current practices of HMA and individualized fortification in neonatal intensive care units (NICUs) across the United States (US) and to inform future translational research efforts implementing this nutrition management method.

**Methods:** An institutional review board (IRB) approved survey was created and collected data on the following subjects such as NICU demographics, feeding practices, HM usage, HM fortification practices, and HMA practices. It was distributed from 10/30–12/21/2020 *via* online pediatric nutrition groups and listservs selected to reach the intended audience of NICU dietitians and other clinical staff. Each response was assessed prior to inclusion, and descriptive analysis was performed.

**Results:** About 225 survey responses were recorded during the survey period with 119 entries included in the analysis. This represented 36 states and Washington D.C., primarily from level III and IV NICUs. HMA was reported in 11.8% of responding NICUs. The most commonly owned technology for HMA is the Creamatocrit Plus TM (EKF Diagnostics), followed by the HM Analyzer by Miris (Uppsala, Sweden). In NICUs practicing HMA, 84.6% are doing so clinically.

**Discussion:** Feeding guidelines and fortification of HM remain standard of care, and interest in HMA was common in this survey. Despite the interest, very few NICUs are performing HMA and individualized fortification. Barriers identified include determining who should receive individualized fortification and how often, collecting a representative sample, and the cost and personnel required.

**Conclusions:** Human milk analysis and individualized fortification are emerging practices within NICUs in the US. Few are using it in the clinical setting with large variation in execution among respondents and many logistical concerns regarding implementation. Future research may be beneficial to evaluate how practices change as HMA and individualized fortification gain popularity and become more commonly used in the clinical setting.

## Introduction

Human milk (HM) is the ideal enteral feeding for nearly all infants and offers unique benefits to the very low birthweight (VLBW) infant population. HM is associated with a decreased incidence of several life, threatening complications of prematurity, including late-onset sepsis (1), necrotizing enterocolitis (2), bronchopulmonary dysplasia (3, 4), and retinopathy of prematurity (5). HM consumption has also been associated with increased height z-scores and decreases in suprailiac skinfold thickness at 5.5 years of age (6), which may be suggestive of improved long-term growth outcomes for VLBW infants who receive HM rather than donor human milk (DHM) or preterm infant formula.

While HM is the preferred type of feeding for VLBW infants, it requires fortification to meet the extraordinarily high nutrient requirements of preterm infants (7, 8). HM fortification is a standard of care in the neonatal intensive care units (NICUs) but is typically done by means of standard fortification, with the false assumption of known HM nutrient density. HM is a dynamic substance with different macronutrient compositions between individuals throughout the lactation period of an individual and even within a single session of expression (9–12). This variability, combined with the high nutrient requirements creates a challenge in the clinical setting to appropriately meet the nutritional needs of preterm infants.

Human milk analysis (HMA) allows for individualized fortification of HM as a strategy to better ensure adequate macronutrient and total energy administration to premature infants. However, individualized fortification is not standard of practice in the United States (US) due to the relatively recent availability of HMA technology for clinical use. At present, only one device for macronutrient analysis has U.S. Food and Drug Administration (FDA) approval for use in the clinical setting; previous use of HM analyzers in the NICU has only been as a part of an institutional review board (IRB) approved research protocol.

## Aim

The aim was to identify current practices of HM analysis and individualized fortification in NICUs across the US and to inform future translational research efforts of implementation of this nutrition management method.

## Materials and Methods

A survey was created and administered using Research Electronic Data Capture (REDCap) tools hosted at Case Western Reserve University. REDCap is a secure, web-based software platform designed to support data capture for research studies. The MetroHealth Medical Center IRB gave ethical approval for this study (IRB 20-00413). A waiver of consent was obtained for all survey participants *via* the REDCap survey.

The survey was piloted within the Ohio Neonatal Nutritionists group (as shown in Table 1) to ensure user-friendly survey design and minimize measurement error. The survey collected data on the following subjects such as NICU demographics, typical feeding practices, HM usage, HM fortification practices, and HM analysis practices. The complete survey can be viewed in Appendix X.

**Table 1:** Description and membership of selected groups and listservs.

**Group**	**Description**	**Membership as of November 2020**
PEDI-RD	Pediatric nutrition listserv hosted by the University of Iowa	2,735
NICU Dietitians	Private Facebook group for NICU Dietitians	693
PNPG Group	The Pediatric Nutrition Practice Group of the Academy of Nutrition and Dietetics	2,751
Ohio Neonatal Nutritionists	Organization of NICU Dietitians in Ohio who practice at a Level III or IV NICU	38

The survey was distributed from 10/30–12/21/2020 *via* online pediatric nutrition groups and listservs selected to reach the intended audience of NICU dietitians and other clinical staff. Groups and listservs were selected to minimize sampling error and reach a large, diverse group of NICU dietitians and clinicians within the US. As shown in Table 1 for a full description of groups.

Study data were recorded in REDCap and downloaded for descriptive analysis, and each response was assessed prior to inclusion in the analysis. Surveys were included if responses had complete demographic information and partial responses for the remainder of the survey. The recorded city, state, and name of the hospital were used to identify duplicate responses from institutions. In instances of duplicate responses, the more complete entries were retained. If multiple complete entries were present, the entries were compared, and agreeing information was retained. If there was conflicting information in the comparison then: reported estimated daily NICU censuses were averaged; if one person entered a value of “didn't know” and one person entered a known value, the known value was used; If one individual indicated that a product was being used while the other did not, it was assumed that the product is being used; a value of “NA” was entered for all other conflicting answers.

## Statistics

Survey data were stored in a secure study database and imported to R Statistical Software (version 4.0.2) (13) for analysis. Descriptive summaries are presented using median [interquartile range (IQR)] or mean (SD) as appropriate for continuous variables and number (%) for categorical variables.

## Results

**Figure 1:**
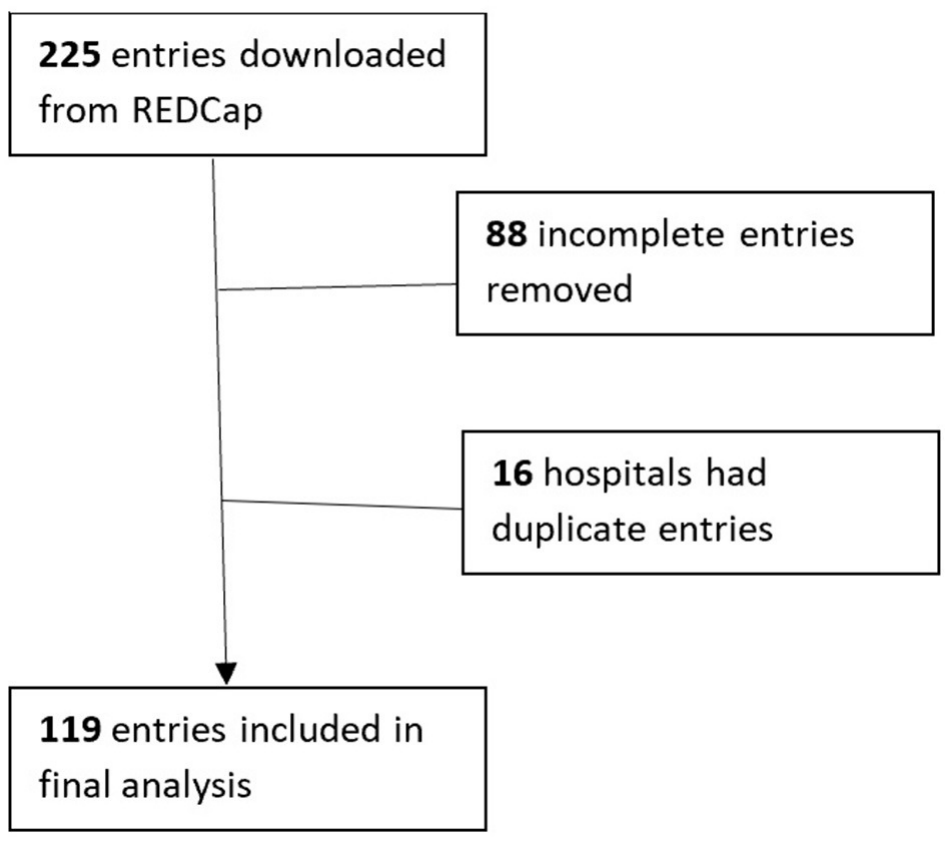
Inclusion of survey responses.

**Figure 2:**
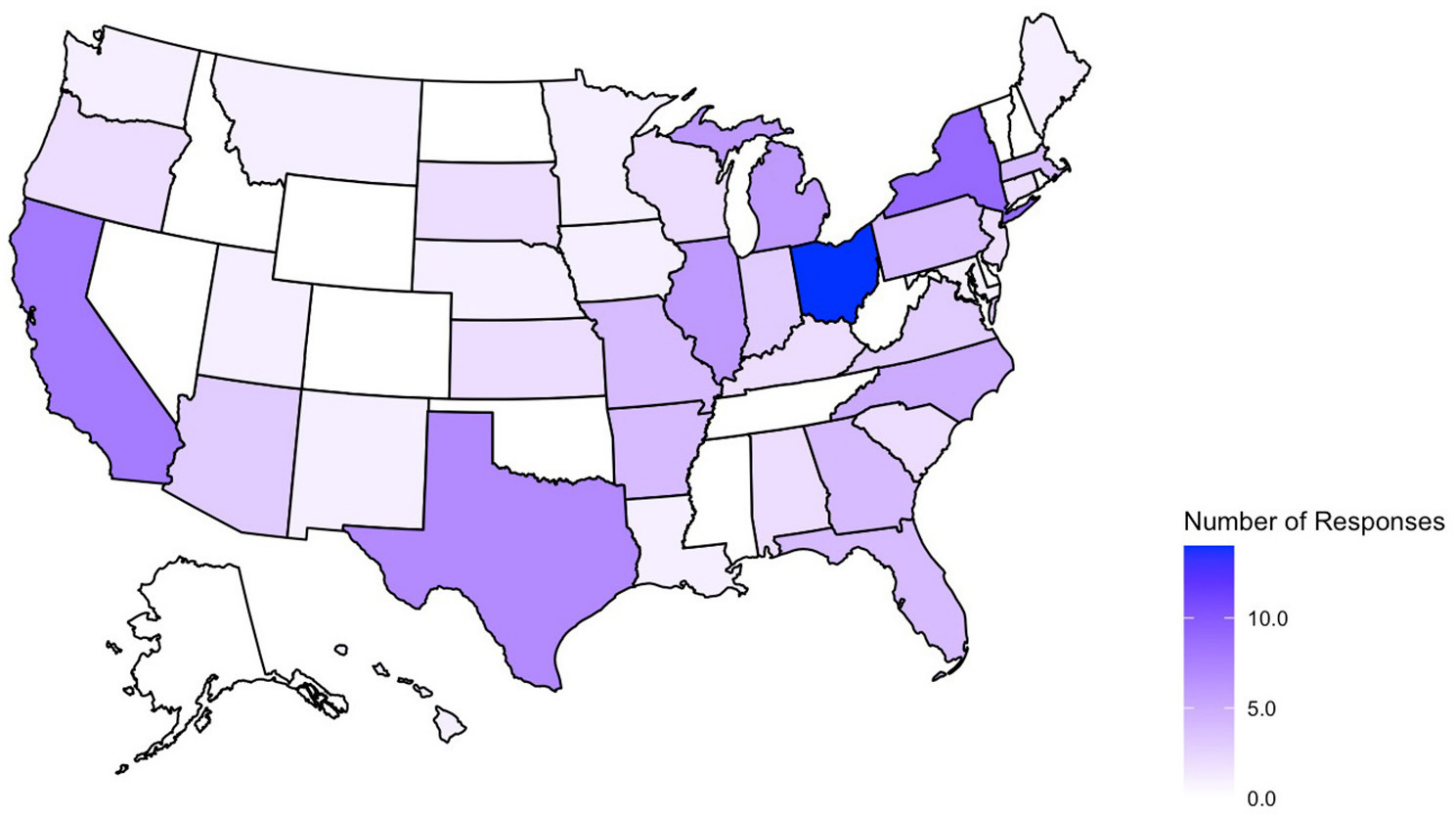
States represented in collected survey responses. No submissions from AK, CO, DE, ID, MS, NV, NH, ND, OK, RI, TN, VT, WV, WY.

A total of 225 survey responses were recorded during the survey period with 119 entries included in the final analysis (as shown in Figure 1). Survey responses were recorded from 36 separate states and Washington D.C (as shown in Figure 2) and represented mostly level III (62.2%) and level IV (35.3%) NICUs. Additional NICU demographic information is displayed in Table 2.

**Table 2:** Demographic information of survey responses.

**Variable**	**Responses (*n* = 119)**
**NICU Type – no. (%)**	
Level 1	1 (0.8)
Level 2	2 (1.7)
Level 3	74 (62.2)
Level 4	42 (35.3)
Children's Hospital – no. (%)	46 (38.7)
Estimated daily NICU census – median (IQR)	35 (25–56)
Survey respondent's role, dietitian – no. (%)	104 (87.4)

**Figure 3:**
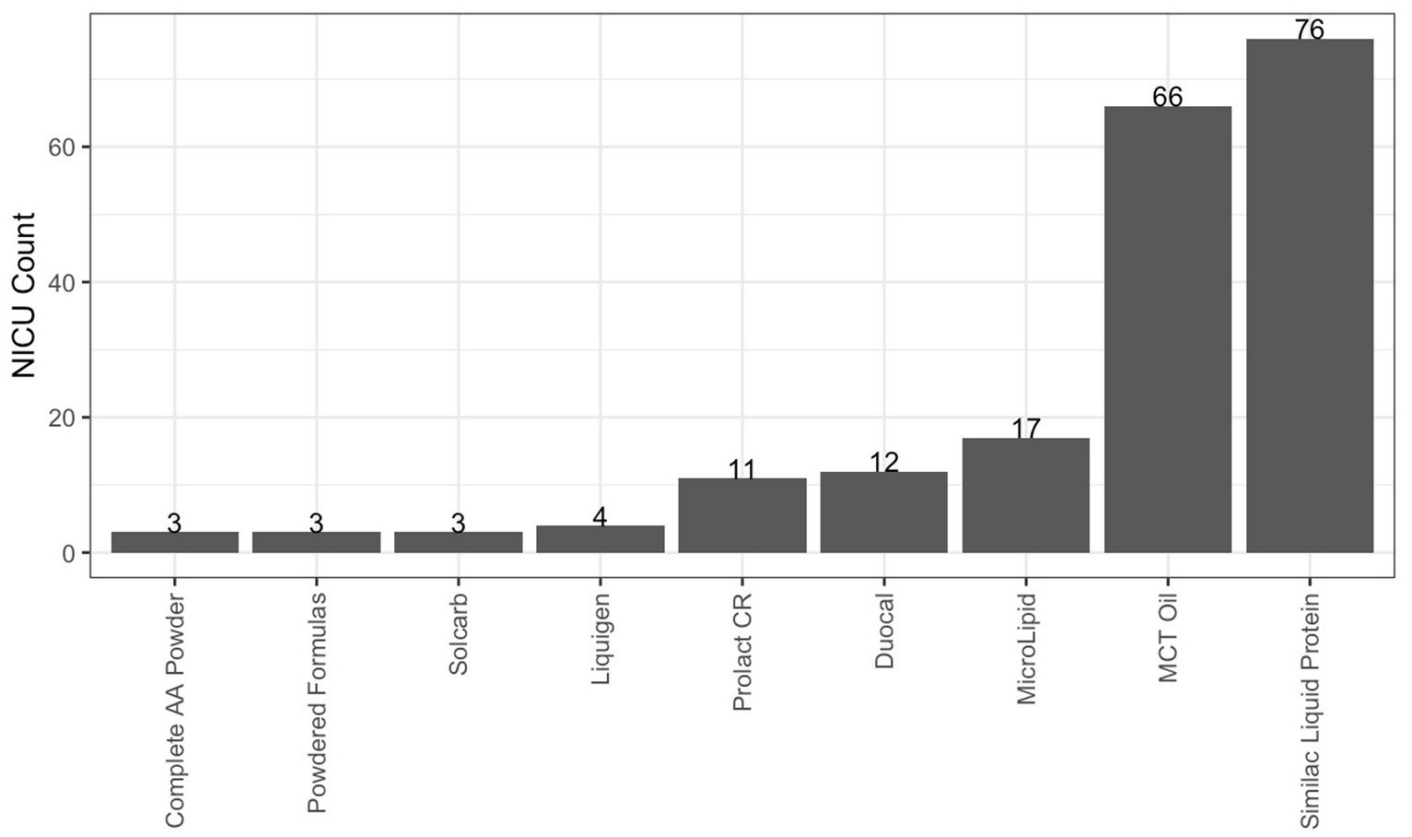
Macronutrient modulars used in responding NICUs. Other products used in fewer than 3 NICUs: canola, oil, fish, oil, olive oil, DHA+ARA, Beneprotein and concentrated liquid formulas.

As described in Table 3, 93.3% of responding NICUs utilize a standardized feeding guideline. A total of 98.2% of responding NICUs utilize some macronutrient modular product in addition to HM fortifiers in their practice. The types of macronutrient modular products used in responding NICUs are described in Figure 3. Modular products are defined as products that provide additional nutrient components to HM, by increasing the amount of protein, carbohydrate, fat, or a combination of macronutrients. Adjustable fortification, the practice of using blood urea nitrogen (BUN) levels to adjust added protein to enteral feeds, was reported in 41.3% of responding NICUs.

**Table 3:** Human milk (HM) fortification and analysis practices.

**Fortification practices among responding NICUs**.	
% Using a standardized feeding guideline	93.3% (111/119)
% Using BUN to individually fortify HM (adjusted fortification)	41.3% (45/109)
% Using macronutrient modular products in their nutrition administration	98.2% (108/110)
**Human milk analysis (HMA) practices among responding NICUs**.	
% Performing HMA	11.8% (13/110)
Of the NICUs performing HMA, % that are doing so in a clinical setting (i.e., not for research)	84.6% (11/13)
**Human milk analysis practices among responding NICUs using HMA clinically**.	
% NICUs with a guideline for performing HMA	25% (2/8)
**Fortification practices**	
Standard	87.5% (7/8)
Individualized – Targeted	0% (0/8)
Individualized – Adjusted	12.5% (1/8)
**HMA frequency**	
As needed	75% (6/8)
More than once weekly	12.5% (1/8)
Weekly	12.5% (1/8)
**HMA sample collection practices**	
A representative sample from a 24-hr period	50% (4/8)
Sample from a single pumping session	37.5% (3/8)
Unknown	12.5% (1/8)

**Figure 4:**
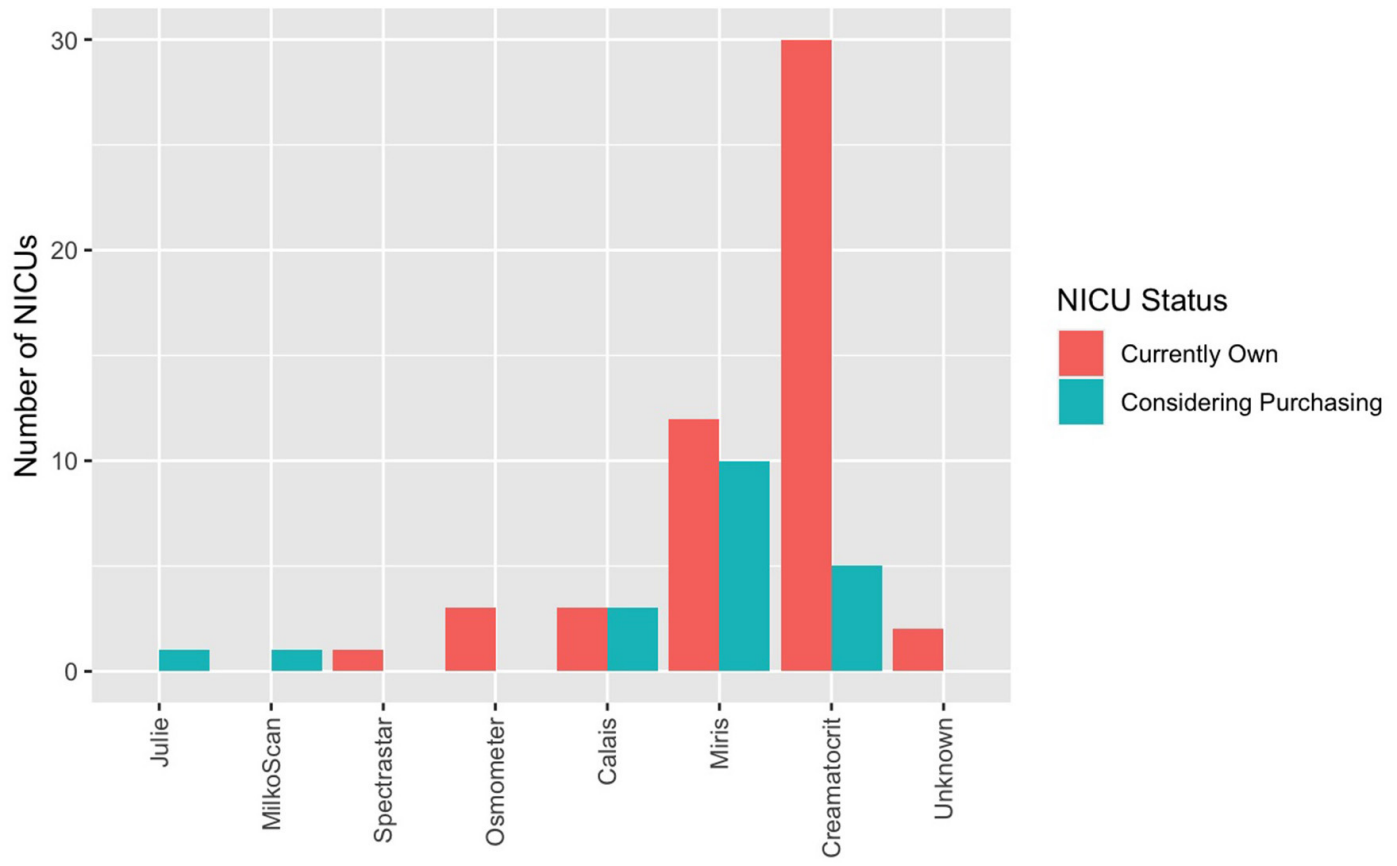
Human milk analyzers in NICUs. What unit currently own (for research or clinical use) and what they are considering purchasing.

**Figure 5:**
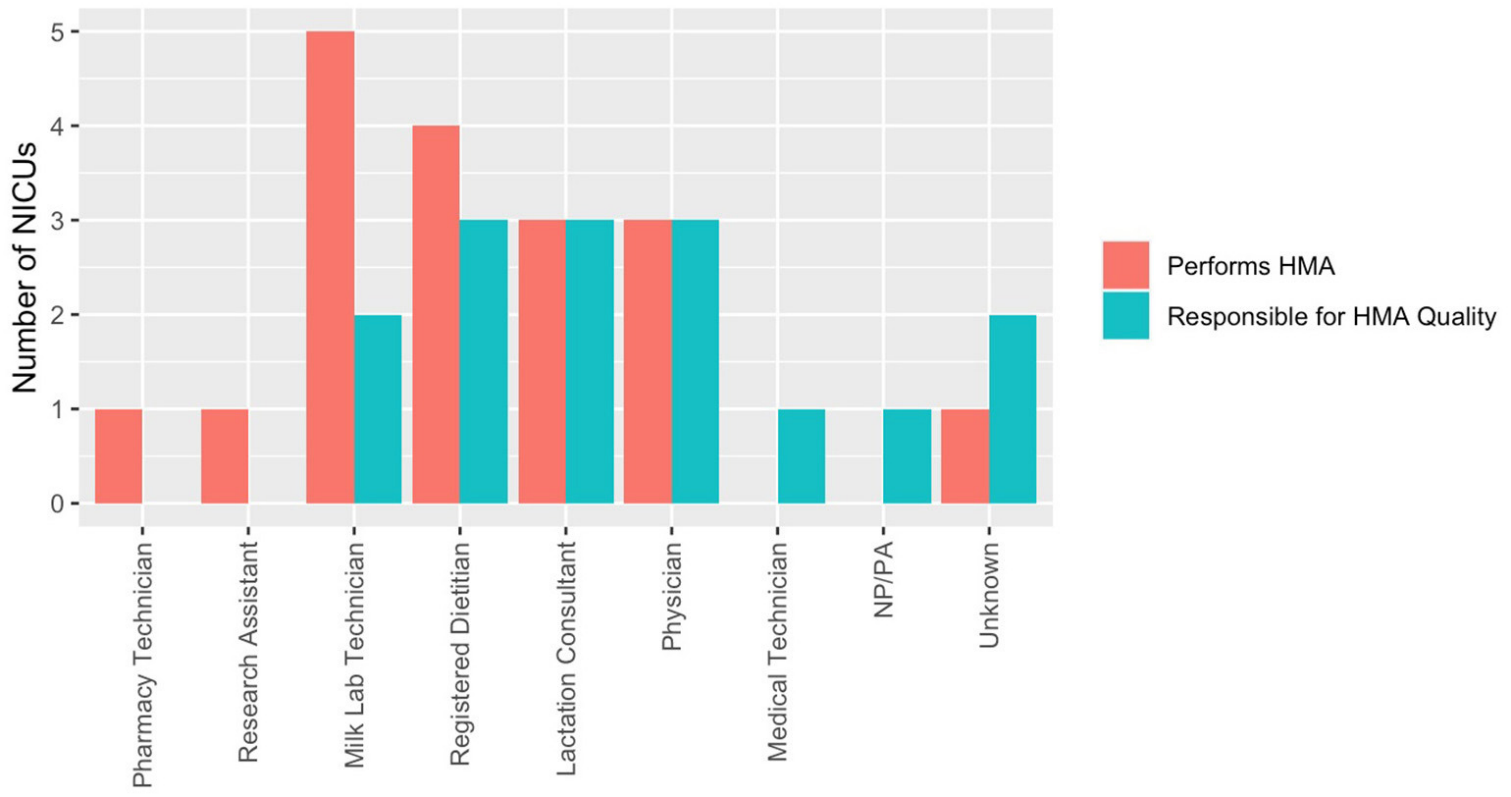
Human milk analysis responsibilities in NICUs. Who performs HMA and who establishes quality of measurements.

Human milk analysis was reported in 11.8% of responding NICUs. The most commonly owned technology for HMA is the Creamatocrit Plus^TM^ (EKF Diagnostics, Boerne, TX, USA), followed by the HM Analyzer by Miris (Uppsala, Sweden) (Figure 4). The most commonly reported HM analyzer that NICUs reported considering purchasing was the HM Analyzer by Miris (Uppsala, Sweden) (Figure 4). The roles of individuals who typically performed HMA and maintained HMA quality in the reporting NICUs are described in Figure 5, while a wide variety of medical professionals perform milk analysis, the responsibility of maintaining the quality of HMA measurements fell largely to registered dietitian nutritionists, lactation consultants, or physicians.

**Figure 6:**
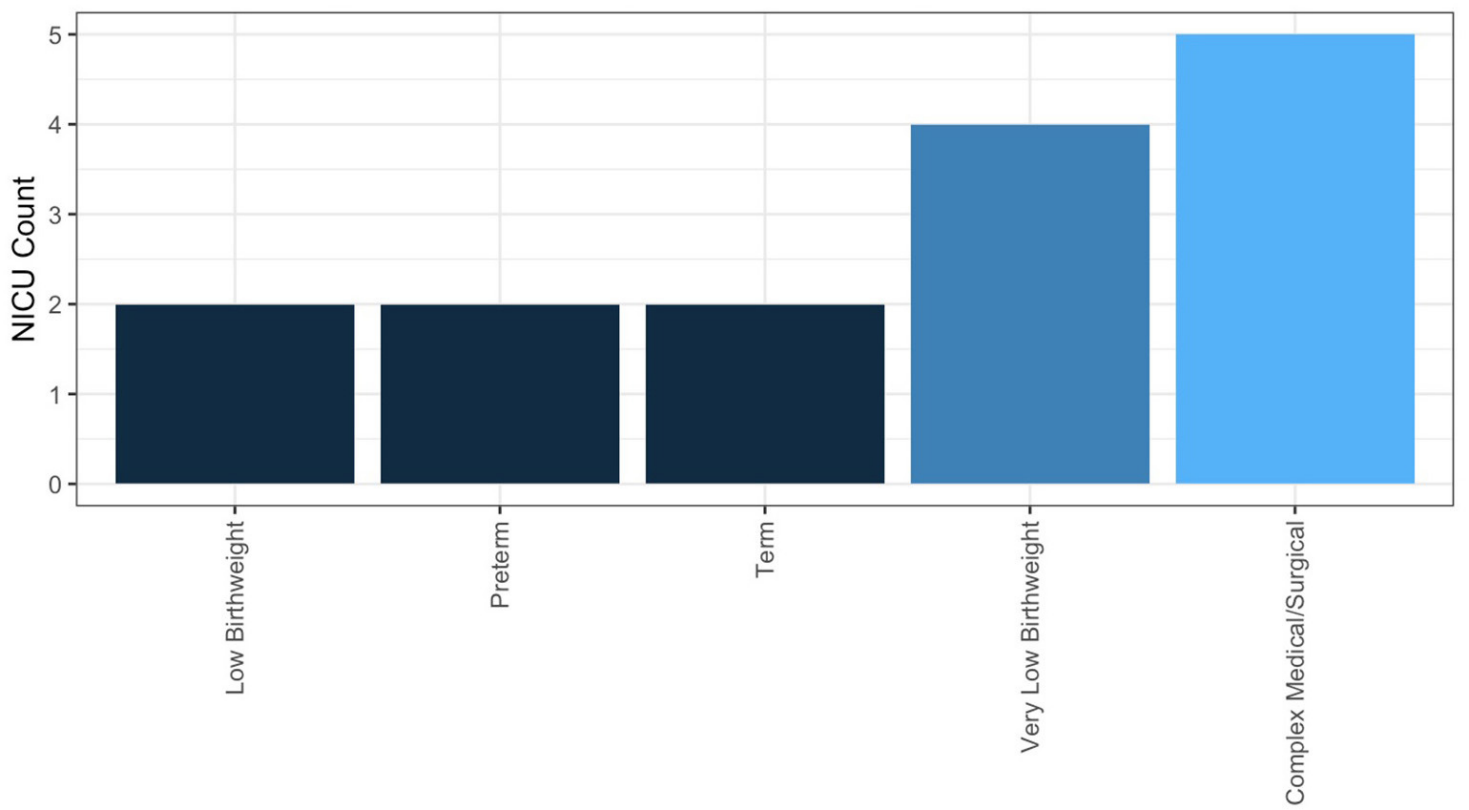
NICUs are using human milk analysis in a wide range of infant populations.

In NICUs practicing HMA, 84.6% are doing so clinically, meaning they are using HMA in the NICU, not under an IRB-approved research protocol. The remainder practice HMA for research alone; 25% of these NICUs having a guideline for performing HMA. Descriptive statistics for frequency of HMA, method of HM sample collection, and fortification practices for NICUs performing HMA clinically are reported in Table 3. Populations, where HMA is being used clinically, are described in Figure 6. How HMA is being used clinically was captured *via* open-text responses and is displayed in Box 1.

Box 1 **Summary of text responses describing how human milk analysis (HMA) is being used in the clinical setting of respondents.**

**Table:** 

**Units using the creamatocrit method**	**Units using mid or near-infrared spectroscopy**
“As a guide as we do not feel it is accurate” “Mainly looking for kcal/oz… if infant isn't growing well” “We rarely have lactation spin a sample and when they do it really doesn't see all that accurate. How can a baby be growing poorly if mom's milk comes back at 27 or 32 cal/oz? It doesn't make sense, so we really don't use it as a general practice.” “to determine net kcal/oz to give to patient”	“[To] increase fortification level for infants with poor growth or decrease if gaining to much weight” “If growth is faltering and we need help adjusting fortification. I do not find the analysis is to be very representative of what the growth chart shows, so we don't do this often” “Based on result, standard fortifier added per our feeding guidelines and then adjusted to meet targeted fortification intake goal. Adjust protein first, then fat and last CHO to meet [estimated] energy goal.” “We use human milk analysis as determined clinically necessary for medically or surgically complex patients to determine macronutrient composition and add a protein modular and fat modular to adjust content as needed.”

## Discussion

Utilization of feeding guidelines and HM fortification remains a standard of care in US NICUs, as evident in the survey responses. There is an increase in interest in individualized fortification methods, and the use of macronutrient modular products in enteral feed administration was common in this survey. HM analysis and individualized fortification are emerging practices within NICUs in the US, with large variation in execution among respondents to this survey. While these practices have been widely discussed in a clinical research setting, translating HMA and individualized fortification into the clinical setting will require implementation research to better evaluate barriers to practice utilization.

### The Rationale for Individualized Fortification/HMA

The known variability of HM is a challenge in creating nutrition plans in the NICU environment, where preterm infants have exceptionally high nutrient requirements that cannot be met by HM alone. Even with standard fortification practices, estimated nutrient requirements may not be met due to the variability of HM (14). HM composition varies both within and between lactating individuals and is influenced by postnatal time and potentially degree of prematurity. Zachariassen et al. (15) found that HM samples were higher in fat and energy content for infants <28 weeks gestational age (GA) compared to those of 28–32 weeks GA (15). Decline in protein content in HM in the first weeks of life has been repeatedly reported (9, 12, 15), although the influence of prematurity on protein content is debated in the published literature (12). Trends of lactose and carbohydrate concentrations within HM have been described in early milk expression (9). However, carbohydrates remain the least accurate and arguably an invalid macronutrient measured using infrared (IR) HM analyzers available for clinical use (16, 17). In a longitudinal study of HM-fed very preterm infants, Belfort et al. (18) found substantial variation in intakes of protein and energy. This variation predicted slower weight gain and linear growth, despite standard fortification in HM-fed very preterm infants. This finding supports the hypothesis that providing individually targeted HM fortification may reduce macronutrient deficits and improve physical growth (18).

Incorporation of the known variabilities in HM into standard NICU nutritional care is a necessary evolution of practice to meet the nutrition requirements of preterm infants. Multiple individualized fortification strategies have been used to address this variability and nutrient requirements, described in Table 4.

**Table 4:** Methods of fortification of HM.

**Method**	**Definition**
Standard Fortification	Addition of fixed doses of commercial human milk fortifiers (HMF) to HM to reach an assumed macronutrient composition
Individualized Fortification	Fortification of HM that is tailored to an individual clinical scenario to improve growth and better meet nutrition requirements (19). Adjustable and targeted fortification are both individualized fortification methods.
Adjustable Fortification	Protein intake is adjusted based on the infant's metabolic response as assessed by BUN levels to improve growth and better meet protein requirements (20). Protein modulars are used in conjunction with HMF in this approach.
Targeted Fortification	Human milk analysis is used to guide fortification practices utilizing HMF and macronutrient modulars to improve growth and better meet energy and macronutrient requirements (21, 22).
Adapted Protein Supplementation	The novel method of individualized fortification that supplements protein based on calculated HM protein values obtained from a validated equation (23).

A recent double-blind randomized control trial by Rochow et al. (21) with very low GA infants fed target fortified HM showed that target fortification of HM with low macronutrient content enhances the quality of nutrition and growth of preterm infants. Not only did they show that target fortification improved weight gain but also had a positive impact on increased body composition, length, and head circumference (21).

Responses to the survey indicated that most NICUs utilize standard fortification practices, using assumed energy and macronutrient density values when adding HM fortifier to HM. However, nearly all (98.2%) responding NICUs reported using at least one macronutrient modular in their clinical practice, with the most commonly reported products such as Liquid Protein Fortifier (*n* = 76, Abbott Nutrition), MCT Oil (*n* = 66, various), MicroLipid^TM^ (*n* = 17, Nestlé Health Science), Duocal (*n* = 12, Neocate), and Prolact CR® (*n* = 11, Prolacta Bioscience). The common utilization of additional macronutrient modulars shows that individualized fortification is already a common, although not standard, practice in US NICUs and one that is driven by clinical assessment, rather than HMA.

The quality of early life nutrition administration may be predictive of body composition development in the preterm infant period (24). Individualized fortification may be a strategy to improve the quality of growth and body composition development in VLBW infants. Parat et al. (25) used HMA and individualized fortification to target a goal of 4 g/kg/day protein intake found that patients who received this targeted fortification for at least 30 days had increased fat-free mass at discharge (25). Morlacchi et al. (26) found that VLBW infants receiving targeted fortification HM had an increased fat-free mass compared to VLBW infants receiving preterm formula at term corrected age (26). While it has not been fully elucidated what early life nutrition interventions promote the best body composition outcomes in preterm infants, the goal of body composition development is becoming clearer. Increased lean body mass in preterm infants is increasingly associated with improved neurodevelopmental outcomes at 4 months (27), 1 year (28, 29), and 4 years of life (30). The potential to improve growth and body composition development in preterm infants is driving the incorporation of HMA into standard NICU care. However, HMA remains in the early phase of clinical implementation in US NICUs, as evidence that only one in 10 responding NICUs is performing HMA clinically.

### Variation in the Method of Analysis

Several methods have been developed to analyze HM for use in individualized fortification and are described elsewhere (31). The two most commonly owned pieces of equipment in the surveyed population were the Creamatocrit Plus^TM^ (*n* = 30) followed by the HM Analyzer by Miris (*n* = 12).

The Creamatocrit Plus^TM^ (EKF Diagnostics) uses centrifugation to estimate the fat concentration and energy density of HM samples. Variations in measured fat content comparing the creamatocrit method to the gold standard gravimetric (or Mojonnier) method have been described in the literature; however, this method remains popular due to ease of use in clinical settings (32, 33). O'Neill et al. (34) found that the creamatocrit method reported mean fat content on average 46% higher and mean energy content 16% higher compared to gold standard analysis *via* the Mojonnier method (34). The same study found that the estimated mean fat content was 80% higher, and the mean energy content was 26% higher compared to milk analysis *via* mid-infrared spectroscopy (Calais HM Analyzer, Metron Instruments, Solon, OH) (34).

The differences in estimated compared to actual energy content could be related to variations in sample collection methods or due to limitations of the creamatocrit method. Concerns for the reliability of this method of HM analysis were noted by two of the NICUs that have access to this technology (as shown in Box 1).

The concerns for the reliability of analysis and the potential insufficiency of assessing only the fat and calorie composition of HM make mid-infrared spectroscopy analysis appealing for clinical use. The HM Analyzer by Miris measures macronutrient concentrations and estimates energy density within a 3 ml HM sample using mid-infrared spectroscopy; this technology was approved for clinical use by the FDA in December 2018. The Miris is the only multi-macronutrient analyzer that is currently approved for clinical use by the FDA and has been validated for macronutrient analysis in numerous studies (17, 35–37). This method of HMA does have significant barriers for many NICUs: it requires a representative sample for accurate analysis, it is an expensive piece of equipment to purchase and requires trained personnel to analyze the milk. Among the surveyed NICUs, the cost was a frequently mentioned barrier for the implementation of HMA.

An additional critical difference between the two most commonly owned HM analyzers is the type of information obtained from their analysis. The Creamatocrit Plus^TM^ provides fat content and an estimated energy density of the milk sample. The Miris provides carbohydrate and protein content, in addition to the fat and energy density of measured milk samples. While there is no evidence that routine fat or carbohydrate supplementation alone improves growth (38, 39), ensuring adequate protein fortification has been associated with improved growth outcomes in preterm infants (38). Combining adequate protein and energy fortification in preterm infants is critical in optimizing growth outcomes. Relying solely on energy density to base individualized fortification decisions is likely not an effective method of improving growth outcomes in preterm infants (40, 41); again, as shown in Box 1.

### Barriers to Clinical HMA Implementation

While the inadequacies of standard fortification methods have been identified, and the interest in individualized fortification is clear, there remain several barriers to the clinical implementation of HMA and individualized fortification in the typical NICU.

#### Barrier 1: Who Should Receive HMA and Individualized Fortification and How Often?

A systematic review and meta-analysis by Fabrizio et al. (42) determined that there was moderate- to low-certainty evidence supporting that individualized fortification (encompassing both targeted fortifications using HMA and adjustable fortification) for VLBW infants improved short-term growth compared to VLBW infants receiving standard fortification (42). Individual studies using HMA for targeted fortification have shown that this practice may improve growth and body composition outcomes (25, 43), but this finding is not universal (44). A lack of clarity in the literature on who individualized fortification would most benefit is reflected in the responses of the survey. From responding NICUs performing HMA clinically (*n* = 11), the majority (*n* = 6) perform HMA for infants that have complex medical or surgical conditions, including infants with poor growth of unclear etiology. Similarly, the frequency of HMA for an individual patient was typical as needed (*n* = 6); a total of two NICUs reported performing HMA at least weekly for an individual patient. Future research exploring both which NICU populations and what outcomes individualized fortification may most benefit from is required to help guide clinical implementation of this practice.

#### Barrier 2: Collecting a Representative Sample

Responses from the survey indicated a variety of sample collection practices for HMA, varying between single expression and 24-h sample collections. Similarly, procedures for sample collection intended for HMA vary in the published literature (45). The method of HM sample collection in a clinical setting must take into account the feasibility of sample collection for the lactating parent and NICU staff (46). However, the influence of HM sample collection on macronutrient composition must be considered when utilizing HMA for clinical nutrition decision-making. The fat content of HM is known to vary most significantly throughout the day and within a single feed (47–49). Protein content is known to decrease over multiple weeks and has also been reported to vary throughout 24 h (11, 15, 50). These macronutrient variations limit the efficacy of using a single feed milk sample for HM analysis in a clinical setting. Obtaining a representative sample from a pooled 24-h sample is essential to accurately capture the average macronutrient and caloric density of an HM sample to create clinical nutrition interventions (16, 51). Failure to obtain a representative sample and accurately perform HMA can drastically change results, fortification administered, and in turn, change the quality of the nutrition received. It is important to note that all samples used for milk analysis are discarded following testing, which may be a deterrent; however, the volume is minimized based on the technology used (e.g., Miris uses, 3 ml sample).

#### Barrier 3: Cost and Personnel

The personnel required for clinical implementation of HMA is a significantly greater cost than the technology alone. Personnel is needed to collect a representative sample (46), analyze the milk (16), create the fortification plan, and then actually prepare the individually fortified HM. Actual individualized fortification should be done in a designated feed preparation space away from the bedside and ideally in a milk lab (52).

Roles identified in this survey as individuals responsible for actually performing HMA included milk lab technicians (*n* = 5), registered dietitians (*n* = 4), physicians (*n* = 3), lactation consultants (*n* = 3), pharmacy technicians (*n* = 1), and research assistants (*n* = 1). Roles identified in this survey as being responsible for HMA quality were as follows: registered dietitians (*n* = 3), physicians (*n* = 3), lactation consultants (*n* = 3), and milk lab technicians (*n* = 2). The translation of a novel method of providing nutritional intervention in a clinical setting means that there is a limited framework in US NICUs on how HMA would best function and be afforded. Comparative effectiveness research to assess standard vs. individualized fortification in VLBW infants is needed to provide cost validation for this process in the typical clinical setting.

#### Solutions?

Human milk analysis and individualized fortification have been successfully implemented into clinical settings (43), but the barriers described above have contributed to this remaining an uncommon intervention in standard NICU practice. These barriers may help explain why only 11.8% of responding NICUs are performing HMA at the time of the survey. Research addressing the above translational gaps will improve the feasibility of the implementation of this practice in NICUs. While HMA remains accessible to only a fraction of NICUs, there appears to be an increasing practice of adjustable fortification, which is the practice of titrating protein fortification on top of a standard fortification based on BUN levels (20). A total of 41.3% of respondents to the survey reported utilizing adjusted fortification in their NICU. Adjusted fortification has been recommended by the European Milk Bank Association as a practical method to optimize HM fortification in the NICU (41). However, this method of fortification requires regular blood draws that may otherwise not be indicated. An adapted protein supplementation strategy proposed by Minarski et al. (23) utilizes a breast milk equation to suggest additional protein fortification based on days after delivery (23). These alternative methods of fortification may be more feasible for the typical NICU given current clinical practice in the US.

### Strengths and Limitations

This study provides a general sense of the current practices of HMA and fortification in US NICUs, a practice that is currently transitioning from the research to the clinical stages of implementation. Formally, capturing typical clinical practices during such a transition period is useful in better identifying both implementation research gaps and practical barriers. Identifying the perspectives and practices of individuals utilizing HMA may help institutions considering HMA identify its usefulness and limitations.

One limitation to the study was the small number of responses from NICUs who are performing HMA in a clinical setting. While this could be due to sampling or a nonresponse error, this finding likely reflects how few NICUs are performing HMA in a clinical setting. An additional limitation to this study, as this was a self-administered survey by self-identified individuals, it is possible that the information collected was not entirely reflective of the practices of a single institution. We attempted to minimize measurement error by piloting the survey among a group of qualified NICU dietitians and utilizing internet groups and listservs that are known to be run by NICU dietitians. We selected NICU dietitians as the target population as we believed this group of professionals would be able to most accurately respond to the survey questions due to their unique professional scope. Some surveys were only partially completed, and it is possible that individuals with less standardized practice or content knowledge were more likely to terminate the survey early.

## Conclusions

Human milk analysis and individualized fortification are emerging practices within NICUs in the US, with large variation in execution among respondents to this survey. This survey provides a general sense of current HM use and an assessment of HMA practices as it is gaining popularity and is becoming more accessible. Very few NICUs are using HMA and individualized fortification in the clinical setting. The survey has also identified many barriers and logistical concerns regarding the implementation of HMA and individualized fortification.

Though we received many responses, the results do not represent all US NICUs. Another limitation to the study was the small number of responses from NICUs who are performing HMA in a clinical setting. Future research may be beneficial to evaluate how practices change as HMA and individualized fortification gains popularity and are more commonly used in the clinical setting.

## Data Availability Statement

The raw data supporting the conclusions of this article will be made available by the authors, without undue reservation.

## Author Contributions

SR, SM, and SG-W designed and distributed the survey. SR, SM, KM, and SG-W performed data analysis and interpreted the results. SR and SM drafted the manuscript. All authors read and approved the manuscript.

## Conflict of Interest

The authors declare that the research was conducted in the absence of any commercial or financial relationships that could be construed as a potential conflict of interest.

## Publisher's Note

All claims expressed in this article are solely those of the authors and do not necessarily represent those of their affiliated organizations, or those of the publisher, the editors and the reviewers. Any product that may be evaluated in this article, or claim that may be made by its manufacturer, is not guaranteed or endorsed by the publisher.
