# Supplementary material for: Exploring Innovations in Human Milk Analysis in the Neonatal Intensive Care Unit: A Survey of the United States
Source: Front Nutr. 2021 Sep 3;8:692600. doi: 10.3389/fnut.2021.692600 (PMC8446443; doi:10.3389/fnut.2021.692600)
Supplement: Supplementary file 1 [file Data_Sheet_1.PDF]

Exploring Innovations of Human Milk Use in the NICU

Objective: To obtain a better understanding regarding human milk preparation, fortification, and analysis in the NICU.

IRB #: IRB20-00413
Date Approved: 9/4/2020
Expiration Date: 8/18/2021 11:59 PM

You are being asked to participate in a research study to learn about Human Milk Use in the NICU. Participating in this study is voluntary. Your involvement will include completing a short questionnaire, which will take about 15 minutes. It will include questions about human milk use, feeding practices, and the use of human milk analysis. If you have already participated, let us know, as you cannot participate more than once.

This is a research study and participation is completely voluntary. Some of the questions you will be asked may make you feel uncomfortable. You have the right to skip any questions that you do not wish to answer or to stop your participation at any time.

Although this survey is anonymous, a rare risk of breach of confidentiality exists. Results will not be reported in any way that it would be possible to identify a participant.

There is no direct benefit to you for participating in the study; however, you will help researchers learn more about Human Milk Use in the NICU.

You will not be paid for your participation in this study.

A decision to not participate in this study will not affect your medical care or result in any loss of benefits to which you are otherwise entitled. If you are an employee or student, your decision to participate or not will not impact your employment or student standing.

If you have questions about any part of the study now or in the future or if you wish to communicate concerns or a complaint, you should contact Dr. Stacey Ramey who may be reached at 216-778-5600. If you have any questions about your rights as a research participant, or if you wish to express any concerns or complaints please contact the MetroHealth Medical Center's Institutional Review Board (which is a group of people who review the research to protect your rights) at 216-778-2021.

By completing this questionnaire, you are agreeing to participate in the study. If you do not wish to participate in the study, then simply do not complete the questionnaire. When you are done please click submit at the end of the survey so your responses may be saved.

Demographics

What type of NICU are you currently located in?	◯ Level 1<br>◯ Level 2<br>◯ Level 3<br>◯ Level 4
What is your approximate average daily census in the past year?	__________________________________
In what type of hospital is your NICU located?	◯ Children's hospital<br>◯ Delivery hospital<br>◯ Other
If other, please specify:	____
In order to ensure that we do not receive multiple responses per NICU, what is the name of your hospital?	__________________________________

In order to identify geographic diversity, in which city is your NICU located? ________________________________

What state is your NICU located in?

- ◯ Alabama
- ◯ Alaska
- ◯ Arizona
- ◯ Arkansas
- ◯ California
- ◯ Colorado
- ◯ Connecticut
- ◯ Delaware
- ◯ Florida
- ◯ Georgia
- ◯ Hawaii
- ◯ Idaho
- ◯ Illinois
- ◯ Indiana
- ◯ Iowa
- ◯ Kansas
- ◯ Kentucky
- ◯ Louisiana
- ◯ Maine
- ◯ Maryland
- ◯ Massachusetts
- ◯ Michigan
- ◯ Minnesota
- ◯ Mississippi
- ◯ Missouri
- ◯ Montana
- ◯ Nebraska
- ◯ Nevada
- ◯ New Hampshire
- ◯ New Jersey
- ◯ New Mexico
- ◯ New York
- ◯ North Carolina
- ◯ North Dakota
- ◯ Ohio
- ◯ Oklahoma
- ◯ Oregon
- ◯ Pennsylvania
- ◯ Rhode Island
- ◯ South Carolina
- ◯ South Dakota
- ◯ Tennessee
- ◯ Texas
- ◯ Utah
- ◯ Vermont
- ◯ Virginia
- ◯ Washington
- ◯ West Virginia
- ◯ Wisconsin
- ◯ Wyoming

What is your role in the NICU?

- ◯ Attending
- ◯ Dietitian
- ◯ Fellow
- ◯ Lactation consultant
- ◯ Nurse
- ◯ Nurse practitioner
- ◯ Resident
- ◯ Other

If other, please specify: __________________________________

Feeding Guideline

Do you use a standardized feeding guideline?	◯ Yes<br>◯ No
If YES, for which patients?	◯ All infants<br>◯ All premature infants (< 37 weeks)<br>◯ All LBW infants (< 2500 grams)<br>◯ All VLBW infants (< 1500 grams)<br>◯ Other
If other, please specify:	__________________________________

Source of Human Milk

Definitions:

Human milk: (HM) encompasses both expressed and donor breast milk.

Expressed breast milk: (EBM) human milk from infant's biological parent.

Donor breast milk: (DBM) human milk from a donor milk bank that has been processed (e.g. Holder pasteurization, agitation retort processing) and procured for hospital use.

Informal milk sharing: human milk provided to a patient that is not from the patient's own biological parent or from a donor milk bank

---

What percentage of VLBW infants receive expressed breast milk as the initial feeding?

- ◯ Few (< 20%)
- ◯ Some (20-40%)
- ◯ About Half (41-60%)
- ◯ Most (61-80%)
- ◯ All (> 80%)
- ◯ Don't know

What percentage of VLBW infants have some expressed breast milk still available at discharge?

- ◯ Few (< 20%)
- ◯ Some (20-40%)
- ◯ About Half (41-60%)
- ◯ Most (61-80%)
- ◯ All (> 80%)
- ◯ Don't know

Do you offer donor breast milk?

- ◯ Yes
- ◯ No

What is your primary source of donor breast milk?

- ◯ In-hospital milk bank
- ◯ HMBANA Milk Bank
- ◯ Medolac
- ◯ Ni-Q
- ◯ Prolacta
- ◯ Other
- ◯ Don't know

If other, please specify:

____

Which group(s) of patients receive donor breast milk standardly in your unit? Select all that apply.

- ☐ Term infants
- ☐ Preterm infants (born < 37 weeks)
- ☐ LBW infants (born < 2500 grams)
- ☐ VLBW infants (born < 1500 grams)
- ☐ Other

If other, please specify:

____

Is your NICU planning on using donor breast milk in the future?

- ◯ Yes
- ◯ No
- ◯ Don't know

Do you use donor breast milk macronutrient and caloric density information provided by the supplier to individualize fortification?

- ◯ Yes
- ◯ No
- ◯ Don't know

Do you allow informal milk sharing in your unit?	◯ Yes<br>◯ No<br>◯ Don't know
Do you have a policy for informal milk sharing in your unit?	◯ Yes<br>◯ No<br>◯ Don't know
If you allow informal milk sharing in your unit, please describe infants for which it is allowed:	__________________________________

Feeding Preparation

Where is your human milk stored?

- ◯ Centralized feeding preparation room (human milk ONLY)
- ◯ Centralized feeding preparation room (human milk & formula)
- ◯ NICU
- ◯ Pharmacy
- ◯ Other
- ◯ Don't know

If other, please specify: __________________________________

Where is your human milk usually prepared and fortified?

- ◯ Centralized feeding preparation room (human milk ONLY)
- ◯ Centralized feeding preparation room (human milk & formula)
- ◯ In the NICU, at the bedside
- ◯ In the NICU, at a dedicated milk preparation space not at the bedside
- ◯ Pharmacy
- ◯ Other
- ◯ Don't know

If other, please specify: __________________________________

Who usually prepares your plain human milk feedings?

- ◯ Bedside nurse
- ◯ Diet technician
- ◯ Milk lab technician
- ◯ Pharmacy technician
- ◯ Other
- ◯ Don't Know

If other, please specify: __________________________________

Who usually fortifies your human milk feedings?

- ◯ Bedside nurse
- ◯ Diet technician
- ◯ Milk lab technician
- ◯ Pharmacy
- ◯ Other
- ◯ Don't know

If other, please specify: __________________________________

What type of human milk fortifier is primarily being used?

- ◯ Enfamil Human Milk Fortifier Powder
- ◯ Enfamil Human Milk Fortifier Acidified Liquid
- ◯ Enfamil Human Milk Fortifier Liquid High Protein
- ◯ Enfamil Human Milk Fortifier Liquid Standard Protein
- ◯ Similac Human Milk Fortifier Powder
- ◯ Similac Human Milk Fortifier Concentrated Liquid
- ◯ Similac Human Milk Fortifier Hydrolyzed Protein Concentrated Liquid
- ◯ Prolacta +4 H2MF
- ◯ Prolacta +6 H2MF
- ◯ Prolacta +8 H2MF
- ◯ Prolacta +10 H2MF
- ◯ Other
- ◯ Don't know

If other, please specify:

____

What type(s) of macronutrient modular are being used in your unit? (select all that apply)

- ☐ Enfamil DHA & ARA Supplement
- ☐ Liquid Protein Fortifier (Abbott)
- ☐ MCT Oil (Nestle)
- ☐ MicroLipid (Nestle)
- ☐ Nutricia Duocal (Nutricia)
- ☐ Nutricia Polycal Powder (Nutricia)
- ☐ Prolact CR ® Human Milk Caloric Fortifier (Prolacta)
- ☐ Other
- ☐ Don't Know

If other, please specify:

____

Do you use BUN for individualized macronutrient fortification of human milk?

- ◯ Yes
- ◯ No
- ◯ Don't know

Human Milk Analysis

Does your institution own any of the following pieces of equipment? (select all that apply)

- ☐ Creamatocrit
- ☐ Osmometer
- ☐ Lactoscan - (Ultrasound)
- ☐ Milk Analyzer Milkoscope Julie - (Ultrasound)
- ☐ Calais Human Milk Analyzer - (Near and Mid-Infrared Spectroscopy)
- ☐ Delta Lactoscope - (Near and Mid-Infrared Spectroscopy)
- ☐ MilkoScan - (Near and Mid-Infrared Spectroscopy)
- ☐ Miris Human Milk Analyzer - (Near and Mid-Infrared Spectroscopy)
- ☐ Near -IR reflectance analysis (NIRA) - (Near and Mid-Infrared Spectroscopy)
- ☐ Unity Scientific Spectrastar - (Near and Mid-Infrared Spectroscopy)
- ☐ Other
- ☐ No
- ☐ Don't know

If other, please specify:

____

Does your institution plan on purchasing any of the following pieces of equipment? (select all that apply)

- ☐ Creamatocrit
- ☐ Osmometer
- ☐ Lactoscan - (Ultrasound)
- ☐ Milk Analyzer Milkoscope Julie - (Ultrasound)
- ☐ Calais Human Milk Analyzer - (Near and Mid-Infrared Spectroscopy)
- ☐ Delta Lactoscope - (Near and Mid-Infrared Spectroscopy)
- ☐ MilkoScan - (Near and Mid-Infrared Spectroscopy)
- ☐ Miris Human Milk Analyzer - (Near and Mid-Infrared Spectroscopy)
- ☐ Near -IR reflectance analysis (NIRA) - (Near and Mid-Infrared Spectroscopy)
- ☐ Unity Scientific Spectrastar - (Near and Mid-Infrared Spectroscopy)
- ☐ Other
- ☐ No
- ☐ Don't know

If other, please specify:

____

Is your unit currently performing human milk analysis?

- ○ Yes
- ○ No
- ○ Don't know

How is human milk analysis being used?

- ○ Clinical
- ○ Research
- ○ Clinical and Research

Who is trained to perform human milk analysis? (select all that apply)	☐ Dietitian<br>☐ Diet technician<br>☐ Lactation consultant<br>☐ Milk lab technician<br>☐ Nurse<br>☐ Nurse practitioner/Physician assistant<br>☐ Pharmacist<br>☐ Pharmacy technician<br>☐ Physician<br>☐ Other<br>☐ Don't know
If other, please specify:	__________________________________
Who is primarily responsible for quality assurance of human milk analysis?	◯ Dietitian<br>◯ Diet technician<br>◯ Lactation consultant<br>◯ Milk lab technician<br>◯ Nurse<br>◯ Nurse practitioner/Physician assistant<br>◯ Pharmacist<br>◯ Pharmacy technician<br>◯ Physician<br>◯ Other<br>◯ Don't know
If other, please specify:	__________________________________

If human milk analysis is being used for 'Research':

Do you plan on performing human milk analysis for clinical use in the near future?

- ◯ Yes
- ◯ No

If human milk analysis is being used for 'Clinical' or 'Clinical and Research' use:

Definitions:

Standardized fortification: the practice of adding a fixed amount of human milk fortifier per 100 mL of HM to achieve recommended nutrient intakes.

Individualized fortification: encompasses both targeted and adjustable fortification methods.

Targeted fortification: the practice of utilizing HM macronutrient analysis to fortify HM to meet energy and specific macronutrient requirements based on the analyzed sample.

Adjustable fortification: the practice of adjusting protein fortification of human milk feeds, using blood urea nitrogen levels as an indirect method of assessing protein adequacy.

Do you have a guideline for which patients you are performing human milk analysis?

- ◯ Yes
- ◯ No
- ◯ Don't know

For which patient populations are you performing human milk analysis? (select all that apply)

- ☐ Term infants
- ☐ Preterm infants (born < 37 weeks)
- ☐ LBW infants (born < 2500 grams)
- ☐ VLBW infants (born < 1500 grams)
- ☐ Medically or surgically complex infants
- ☐ Other

If other, please specify:

____

Please describe how you are using the results of human milk analysis for these patient populations.

____

For an individual patient, about how frequently are you performing human milk analysis?

- ◯ As determined clinically necessary
- ◯ More than once per week
- ◯ Once per week
- ◯ Every other week
- ◯ Once per month
- ◯ Other
- ◯ Don't know

If other, please specify:

____

How do you usually obtain a sample for analysis?

- ◯ Representative sample from a 24 hour collection
- ◯ From single pumping session
- ◯ Other
- ◯ Don't know

If other, please specify:

____

Please describe the typical procedure(s) for how human milk samples are usually obtained for analysis.

____

Are you routinely providing standard or individualized fortification?	◯ Standard<br>◯ Individualized- Targeted<br>◯ Individualized- Adjustable<br>◯ Other<br>◯ Don't know
If other, please specify:	__________________________________
Which patients are receiving individualized fortification? (select all that apply)	☐ Term infants<br>☐ Preterm infants (born < 37 weeks)<br>☐ LBW infants (born < 2500 grams)<br>☐ VLBW infants (born < 1500 grams)<br>☐ Medically or surgically complex infants<br>☐ Other
If other, please specify:	__________________________________
